# Evaluating Technicians’ Workload and Performance in Diagnosis for Corrective Maintenance

**DOI:** 10.3390/s24061943

**Published:** 2024-03-18

**Authors:** Hyunjong Shin, Ling Rothrock, Vittaldas Prabhu

**Affiliations:** Marcus Department of Industrial and Manufacturing Engineering, The Pennsylvania State University, University Park, PA 16802, USA; hjshin.jacob@gmail.com (H.S.); lxr28@psu.edu (L.R.)

**Keywords:** maintenance, diagnosis support system, human–smart system interface

## Abstract

The advancement in digital technology is transforming the world. It enables smart product–service systems that improve productivity by changing tasks, processes, and the ways we work. There are great opportunities in maintenance because many tasks require physical and cognitive work, but are still carried out manually. However, the interaction between a human and a smart system is inevitable, since not all tasks in maintenance can be fully automated. Therefore, we conducted a controlled laboratory experiment to investigate the impact on technicians’ workload and performance due to the introduction of smart technology. Especially, we focused on the effects of different diagnosis support systems on technicians during maintenance activity. We experimented with a model that replicates the key components of a computer numerical control (CNC) machine with a proximity sensor, a component that requires frequent maintenance. Forty-five participants were evenly assigned to three groups: a group that used a Fault-Tree diagnosis support system (FTd-system), a group that used an artificial intelligence diagnosis support system (AId-system), and a group that used neither of the diagnosis support systems. The results show that the group that used the FTd-system completed the task 15% faster than the group that used the AId-system. There was no significant difference in the workload between groups. Further analysis using the NGOMSL model implied that the difference in time to complete was probably due to the difference in system interfaces. In summary, the experimental results and further analysis imply that adopting the new diagnosis support system may improve maintenance productivity by reducing the number of diagnosis attempts without burdening technicians with new workloads. Estimates indicate that the maintenance time and the cognitive load can be reduced by 8.4 s and 15% if only two options are shown in the user interface.

## 1. Introduction

Advancement of digital technologies enables smart and automated processes. Businesses introduce new technologies to achieve competitive advantages over their competitors by changing tasks, processes, and the ways we work [1]. Maintenance is an especially promising area where adopting new digital technologies can bring great productivity improvements, because currently, only a few maintenance activities are fully automated [2,3,4]. For many maintenance activities dealing with failures and faults, a large portion of maintenance time is used for diagnosis, which is usually a trial-and-error processes. This requires repetitive activities until a technician finds the cause of machine failure [5]. Thus, utilizing a diagnosis support system can improve maintenance productivity, but needs to be studied from different perspectives, especially the effect on users of such diagnosis support systems [1,6]. One study tested the influence of 3D CAD models and virtual reality models on working memory in the construction industry [7]. Recently, a digital twin for cognitive tasks in production and maintenance operations has been proposed for process industries [8]. User acceptance of new technologies such as cognitive assistance using head-mounted devices is being studied [9]. Another related advancement being studied is augmented reality to improve the cognitive load on assembly workers in the automotive industry [10]. Head-mounted displays using augmented reality with animation has been found to improve performance by 14% compared to video instructions [11]. In another study, augmented reality was found to be beneficial in several ways in a mold-making industry [12]. But few studies have been conducted to analyze the effect of the different diagnosis support systems on technicians.

The overall objective of the paper is to address the research gap caused by a dearth of empirical data related to maintenance, which is a barrier to efficiently assessing the effectiveness of various technological advances that may be beneficial for improving maintenance operations. Moreover, in our collaborations with several manufacturing companies, we noted that such data are rarely captured in industry today. As a first step to address this gap in the field of maintenance, this paper embarks on a study to understand and characterize diagnostic time by using the methodology of experimental research. Specifically, the methodology is used to conduct experiments with human subjects to test the following hypotheses:

- H1: The task completion time of the group which uses the AI-based support system will be shorter;
- H2: The cognitive load of the group which uses the AI-based support system will be lower.

The preliminary study was conducted with 10 participants to analyze the effect of the diagnosis support system [13]. This paper is a continuation of our previous work and the effort to design a human centered diagnosis support system for maintenance tasks.

The structure of this paper is as follows. In Section 2, the authors review artificial intelligence (AI) applications in the domain of maintenance. Also, the effects of the system interface on users are reviewed. In Section 3, the experiment which aims to compare the effects of two different diagnosis support systems is presented. In Section 4, the findings using natural goals, operators, methods, and a selection rules language (NGOMSL) model are discussed. Lastly, in Section 5, directions for future research are presented.

## 2. Literature Review

Traditionally, diagnosis tasks are aided by a fault-tree analysis that systematically localizes the cause of a failure mode. With the advancement in digital technologies, alternative approaches using machine learning (ML) and artificial intelligence (AI) are becoming more attractive [14,15,16,17]. Industrial internet of things, cloud technology, and information and communication systems are enablers of such systems [2]. In this section, a summary of related works on two topics is presented: machine learning and artificial intelligence in maintenance and human-centered system interaction. “Google Scholar” is used for the search, and only academic papers such as journal articles, conference proceedings, and dissertations are included. The search key words for the first topic are “maintenance”, “artificial intelligence” and “machine learning”; the search key words for the second topic are “human workload”, “human error”, interaction, “system”, and “artificial intelligence”.

### 2.1. Machine Learning and Artificial Intelligence in Maintenance

Many AI application studies in manufacturing focus on finding the root cause of production defects using different methodologies, such as the implementation of a Bayesian network, artificial neural network (ANN), or support vector machine [14,18]. The industries studied include the semiconductor and the automobile industries [16,19,20]. The other AI application used in the literature is a method to predict the performance of the process by finding errors in the processing stage [21,22]. AI can also be used to diagnose quality issues during or after production. For example, the surface roughness of the processed materials is predicted using an AI approach [23,24].

AI can also be used to deal with machine failures, including corrective maintenance. For example, detecting vehicle faults is one common application [17]. Another use is to detect the degradation of machines and tools or to detect the fault of a machine [25,26,27]. However, AI is widely studied for preventive or condition-based maintenance where AI methods are used to analyze large data sets and real-time data for predicting the condition of a system. For example, the healthy state of a component in a wind turbine has been predicted based on the vibration data [28]. Similarly, the healthy state of an engine has also been predicted based on the vibration data [29]. In addition to the vibration data, temperature measurements from 21 sensors have been used to predict the current condition of an engine [30]. Instead of focusing on the healthy state of a component, AI has also been used to capture and classify failure signatures and associate these with possible failures [31].

Most current studies on applying AI for maintenance predominantly focus on applying new machine learning methods with different data sets for prognostic maintenance, as shown in Table 1. However, its impact on technicians performing diagnoses for corrective maintenance are very limited. The study of the interaction between technicians and diagnostic systems for corrective maintenance is especially rare.

### 2.2. Human-Centered System Interaction

The increased use of digital technology in manufacturing is meant to improve operators’ performance. However, interacting with an inappropriately designed system may act as a barrier and create even more unidentified challenges [32], because many studies focus on the technology and fail to consider the contribution and role of humans [33]. Thus, the importance of human-centered design is emphasized [32,34,35]. For example, Pacaux-Lemoine addressed the importance of designing a system based on human–machine cooperation principles and tested the workloads of the different systems using NASA-TLX (Task Load Indicator) [36]. Cimini stated that the development of new smart technologies should occur together with the developments in the human-related aspects [37].

Depending on the interface and methodology behind a diagnosis support system, technician workload may differ. As more information is stored and transmitted electronically, the workload difference due to the presentation of media is being studied by various methods [38,39,40]. Besides studying the effect of media presentation, other researchers have studied the effect of the interface on performance and workload in selected domains. For example, in one study, the effect of the method of interaction with a mobile application on performance and workload was investigated [41]. In another study, the influence of user interface on performance and situational awareness under a controlled lab environment was experimentally investigated [42]. Other studies have focused on estimating operator workload in highly stressful environments, such as nuclear power plants and shipping ports, using NASA-TLX and the eye-tracking method [43,44,45]. Therefore, this paper takes the first step towards developing a human-centered approach by studying the effect of a smart diagnosis support system for maintenance on technicians in a controlled lab setting.

## 3. Experiment

From the literature reviews, there are very little empirical data on maintenance tasks such as diagnostics and repairs times. However, there is considerable effort underway to make maintenance smarter by deploying advanced technologies [14]. The academic approach to work around this gap in understanding is to lump all such uncertainties in diagnostics and repair times together and assume probabilistic distributions to characterize them. As a first step to address this gap in the field of maintenance, this paper embarks on a study to understand and characterize diagnostic time. A similar approach would also be needed for repair times, but this is outside the scope of the paper.

**Figure 1:**
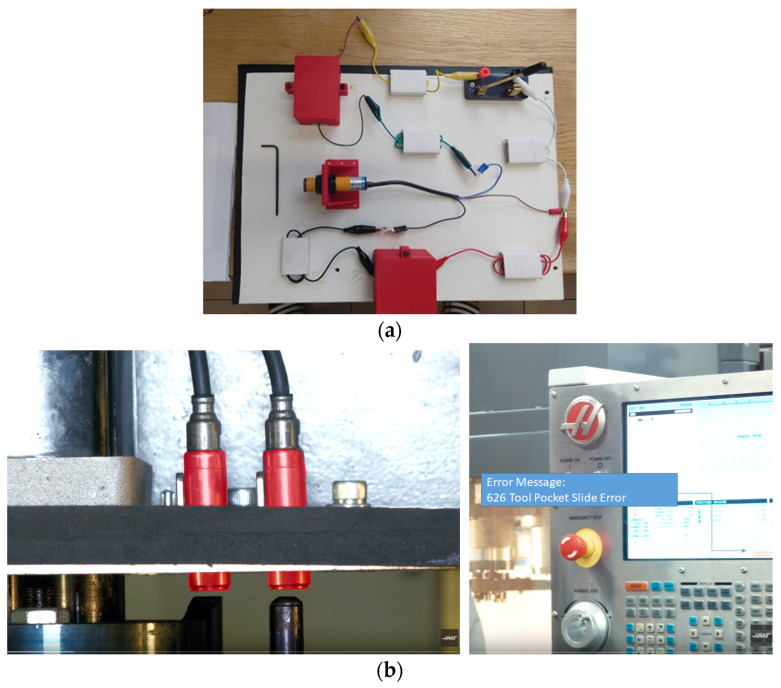
(**a**). Electric circuit model used in the experiment. (**b**). Actual proximity switch on a CNC machine and error message on console.

In this experimental study, the focus is on the maintenance of computer numerical control CNC machine tools, a class of automated machinery widely used in various manufacturing industries. We did not find any publication in the open literature that has studied the interaction between technicians and diagnostic systems for corrective maintenance. Hence, we discussed with field technicians to set up this experiment. One of most frequent failures of automated machines revolves around [46] proximity switches that are used to ensure the proper positioning of the object intended for CNC operation or to verify the presence of safety guards. To repair a proximity switch-related fault, technicians have to diagnose the electrical sub-system of motion control, such as cables, power supply, or input/output board of the controller, and the task is not merely replacing a switch. To translate the diagnosis of proximity switches into a controlled laboratory experiment, an electric circuit model with all the components that are typically involved in the corresponding subsystem of CNC machines is developed as shown in Figure 1. Appendix A includes links to videos that describe proximity sensors and related troubleshooting.

**Figure 2:**
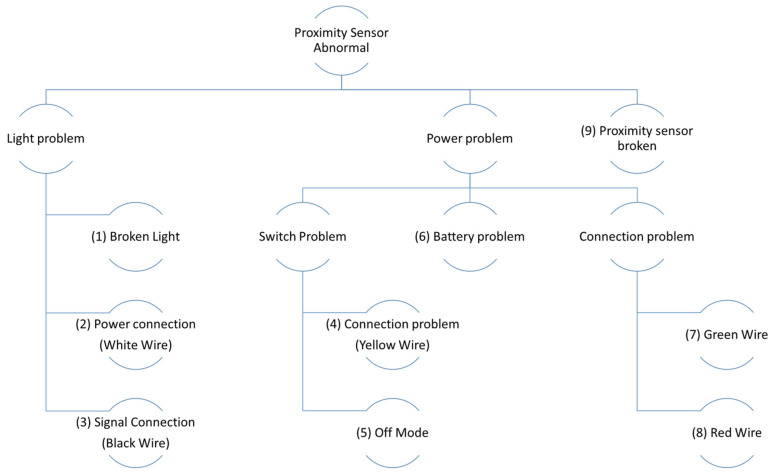
Fault tree analysis for the proximity sensor with nine components.

Repairing and maintaining machines is commonly performed by using FTd-systems. The system supports technicians by identifying the cause of problems using the deductive failure analysis methods. This approach is logical, but the common cause of failure is not obviously identified and may require unnecessary maintenance steps. For instance, there could be nine causes for a fault in the proximity sensor, as shown in Figure 2. If the cause of the fault is red wire, then the technician needs to go through eight components in the FTd-system. Seven of these steps may have been avoided if there were a smarter way to support technicians’ diagnosis.

One smarter way to achieve this is using AId-systems by proving technicians’ component failure probabilities based on historic data and potentially updating these probabilities as the current diagnosis process progresses. The advantage of this approach is that it can identify a common cause of failure first. In addition, this may require fewer maintenance attempts compared to the FTd-system approach, since it can eliminate options or update the failure probabilities as the condition of components is updated by users or sensors. The experimental setting emulated the proximity switch circuit and experimentally evaluated technicians’ performance in the laboratory, thereby avoiding uncontrolled environmental factors.

### 3.1. Experimental Design and Setup

In this subsection, the experimental setup will be described in detail, along with the diagnosis support systems, measurements, and the protocol used.

#### 3.1.1. Experiment Setting

The electric circuit model for this experiment is composed of five cables and one of each of the following: sensor, battery, switch, and light bulb. Every component in the model represents some component in a real industry machine, as shown in Table 2. The proximity sensor in the model detects whether the door is closed and shuts off the power depending on the position of the door.

In the experiment, four components can be intentionally rendered faulty: (1) battery, (2) switch, (3) light bulb, and (4) signal cable to light bulb (black cable). Participants diagnose and repair the model by interacting with either a fault tree diagnosis support system (FTd-system) or an artificial intelligence diagnosis support system (AId-system).

#### 3.1.2. Diagnosis Support System

In practice, the probability for AId-systems can be calculated based on the past maintenance log, data collected from IoT sensors, and the AI algorithms used. The focus here is on the technician’s interaction with the system calculating probabilities; the specific way in which the probabilities are calculated is treated as a black box. For example, based on the condition of each component at the time of the failure, as shown in Table 3, the probability that a particular component is in bad condition can be calculated as shown in Table 4.

**Figure 3:**
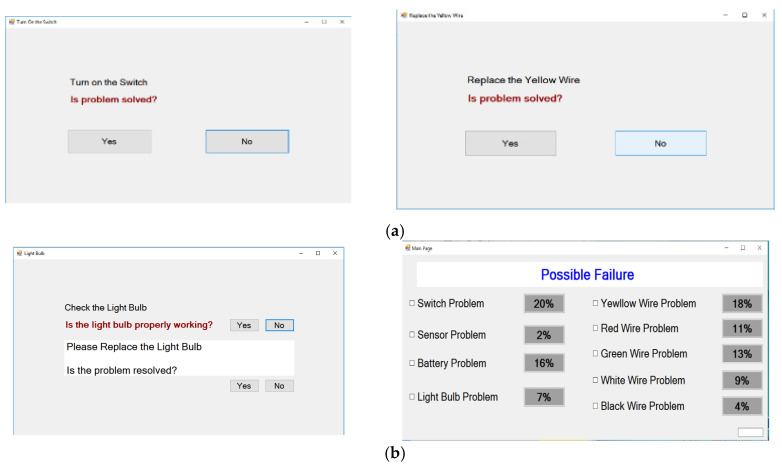
Diagnosis support system interface. (**a**) FTd-system interface. (**b**) AId-system interface.

In this experiment, the probability given by the AId-system is assigned randomly, but in a way that participants can complete the maintenance task in 8 attempts. In an industrial implementation, these probabilities would be calculated using machine learning approach such as a Bayesian network trained using a historic maintenance log. The interface of two systems is as shown in Figure 3.

#### 3.1.3. Measures

To analyze the impact of the diagnosis support system on technicians during maintenance, five measures are used: (1) time to complete, (2) NASA Task Load (NASA TLX), (3) diagnosis attempt, and (4) unnecessary replacement.

- *Time to complete* is the time that a participant takes to diagnose components and replace them. This comprises diagnosis time, such as using a diagnosis support system and a multimeter, and the time to isolate and replace components.
- *NASA Task Load Index (TLX)* is a subjective assessment tool that rates the perceived workload of participants. Participants rate their workload using the rating scales form at the end of the experiment [47,48]. The rates are weighted according to the source of the workload [49].
- *Maintenance attempts* is the number of parts that are maintained by a participant. If the participant strictly follows the suggestion of the diagnosis support systems and makes no errors, the number of attempts should be eight.
- *Unnecessary replacement* is the count of components that are in good condition but were replaced by a participant during the maintenance.

#### 3.1.4. Experiment Procedure

The experiment procedure consists of four sessions: (1) screening and training, (2) preliminary study, (3) study, and (4) post-study.

The screening and training session begins upon the arrival of a participant. The requirements of the experiment are explained to the participant. If the participant meets all requirements of the experiment, a written informed consent form which has been confirmed by the organization’s Institutional Review Board is given to the participant to review. A signature is not collected from the participant, but the participant expresses their implicit consent by participating in the study. Only after reviewing the informed consent form is demographic information such as age and gender of the participant collected. Then, the parts of the electric circuit with the proximity sensor used for the experiment and the ways to diagnose each part with a multimeter are explained to the participant. Furthermore, the support system that the participant will interact with during the study session is introduced. After the explanation, the participant is given as much time as they need to experiment with the electric circuit, the multimeter, and the support system to become familiar with the setup.

After the screening and training session, the preliminary study is conducted. In this session, the basic performance of the participant is measured. The participant is asked to remove the cover, isolate or detach one part from the circuit model, diagnose the condition of the part, put it in the circuit, and put the cover back.

A break time is given to the participant after the preliminary study session if they desire. Then, the same circuit model, but with intentionally rendered faulty components, and the support system is presented to the participant. The study session begins when the participant clicks “Start Diagnosis” in the support system. The study ends when the participant successfully completes maintenance of the model or attempts to diagnose all nine components in the model.

Upon maintenance completion, the post-study session begins as the participant is asked to complete a NASA TLX survey. Then, a monetary award is given to the participant after signing the award receipt. Lastly, the participant has time to raise concerns or ask questions regarding the experiment and is dismissed if they do not have any questions.

#### 3.1.5. Pilot Study

**Figure 4:**
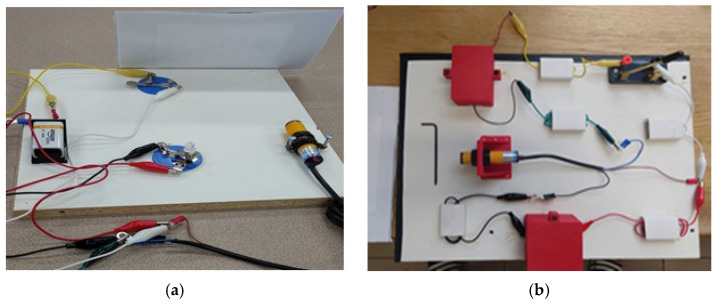
Experiment setting before and after changes. (**a**) Experiment setting before adding covers. (**b**) Experiment setting after adding covers.

With 10 participants, the pilot study was conducted to explore any unexpected issues and to determine the sample size for the experiment. After the pilot study, the covers were added to the top of each component to mimic a practical maintenance task more closely as shown in Figure 4.

#### 3.1.6. Sample Size

With the mean and standard deviation collected from the pilot study, the number of participants for the experiment was determined using power analysis. Since the standard deviation of the preliminary study was 114.83, the experiment required at least 9 participants for each group at a two-tailed 5% level and 80% power.

### 3.2. Experiment Results and Analysis

Thirty adults (mean age = 24.87 years old, range 19–38) participated in the study. All participants were recruited by advertisements posted around the campus. There were 20 male participants and 10 female participants. All the participants were college students with no relevant experience in diagnostics of CNC machine tools or the model used in the experiment (the subjects recruited in the pilot study were forbidden to participate in the experiment). Also, to minimize gender effects, similar numbers of female participants were assigned to each group.

**Figure 5:**
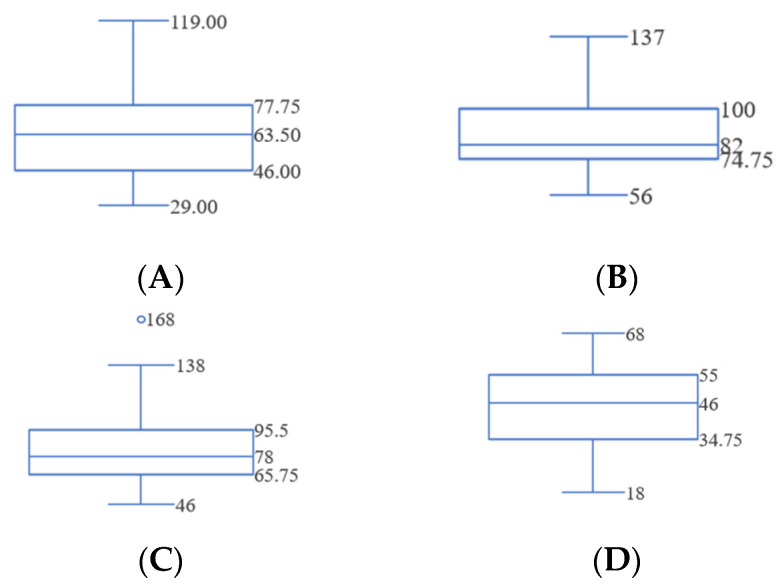
Preliminary study result (units in seconds). (**A**) Sensor diagnosis time. (**B**) Battery diagnosis time. (**C**) Light bulb diagnosis time. (**D**) Wire diagnosis time.

The average times to diagnose the sensor, the battery, the light bulb, and the wire were 66 s, 86 s, 85 s, and 45 s, respectively, as shown in Figure 5.

To see the effects of two different diagnosis support systems, each group’s performance was compared by a two-tailed *t*-test.

#### 3.2.1. Time to Complete

**Figure 6:**
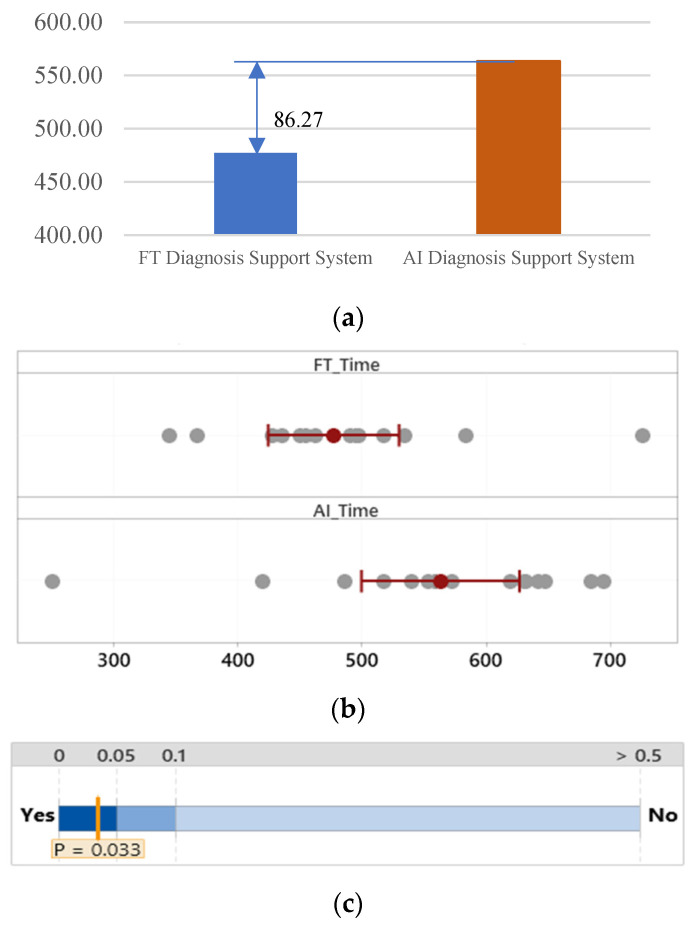
Mean difference in time to complete (units in seconds). (**a**) Mean time to complete difference. (**b**) Two groups’ distributions of data. (**c**) Two-sample mean *t*-test.

The group that used the FTd-system had shorter completion times (*M* = 477.20 s, *SD* = 94.96) than did the group that used the AId-system (*M* = 563.47 s, *SD* = 114.85), *p* = 0.03, as shown in Figure 6. The coefficient of variation of the group that used the FTd-system was 19.90%, and that of the AId-system was 20.38%. The group that used the FTd-system completed the task 86.27 s faster than the AId-system group.

#### 3.2.2. NASA TLX

**Figure 7:**
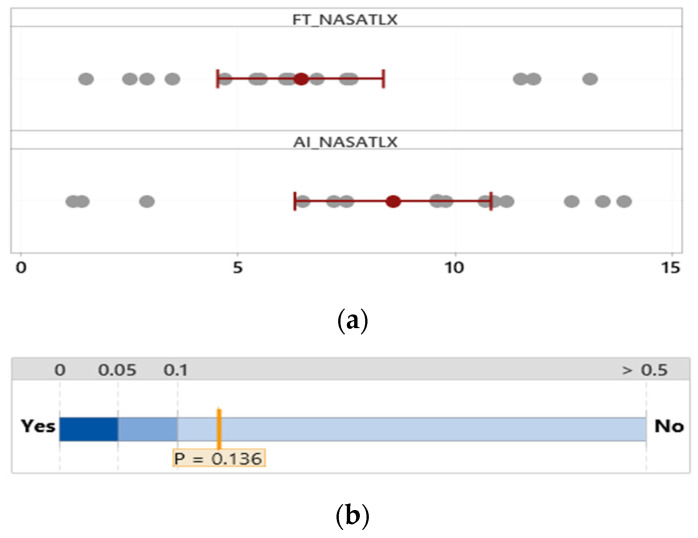
Mean difference in NASA TLX. (**a**) Distribution of data. (**b**) Two-sample mean *t*-test.

**Figure 8:**
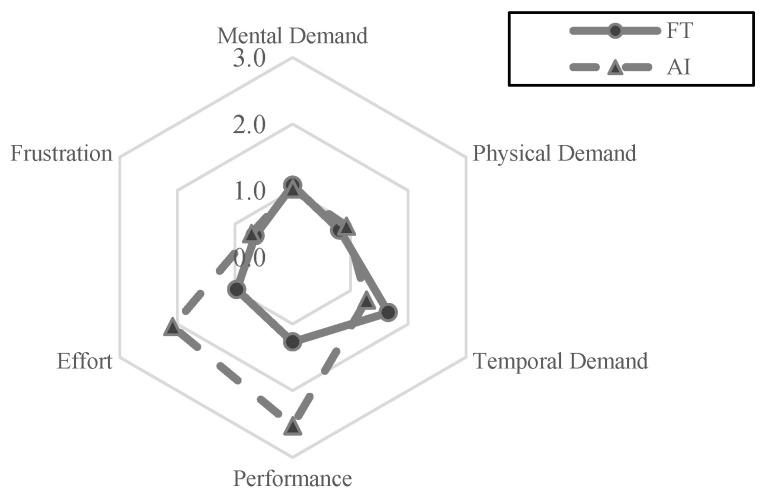
Source of overall workload.

**Figure 9:**
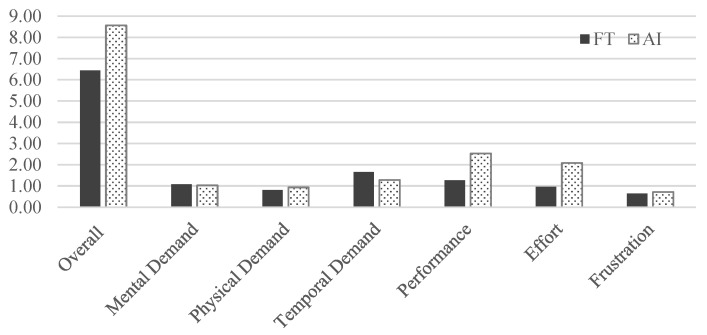
Workload comparison.

The overall workload difference measured by NASA TLX between the group that used the FTd-system (*M* = 6.44, *SD* = 3.35) and the group that used the AId-system (*M* = 8.56, *SD* = 4.09) was indifferent (*p* = 0.14), as shown in Figure 7, although the two major sources of the workload were different depending on the group, as shown in Figure 8 and Figure 9.

#### 3.2.3. Maintenance Attempt

**Figure 10:**
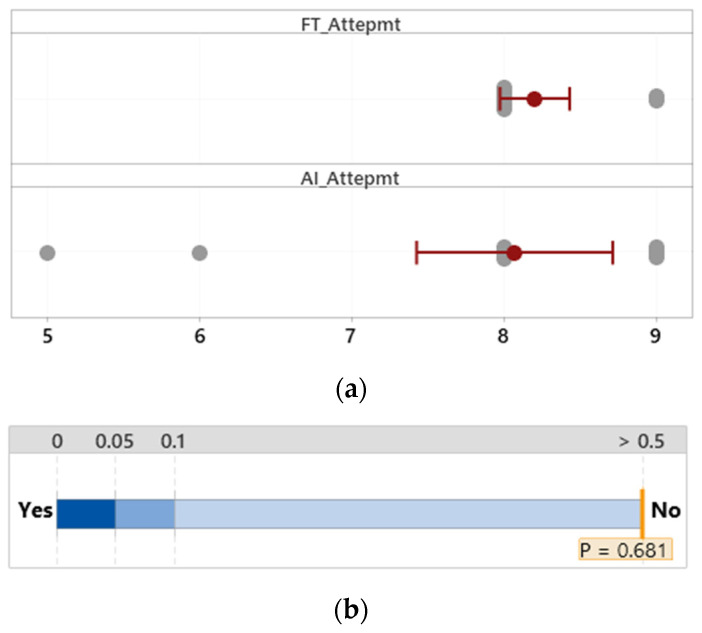
Mean difference in maintenance attempt. (**a**) Distribution of data. (**b**) Two-sample mean *t*-test.

The number of diagnosis attempts for both groups was expected to be 8 if all participants strictly followed the suggestion of the diagnosis support system and made no errors. The difference in the number of diagnosis attempts between the group that used the FTd-system (*M* = 8.20, *SD* = 0.41) and the other group (*M* = 8.07, *SD* = 1.16) was only 0.13 and insignificant, *p* = 0.68, as shown in Figure 10.

#### 3.2.4. Unnecessary Replacement

**Figure 11:**
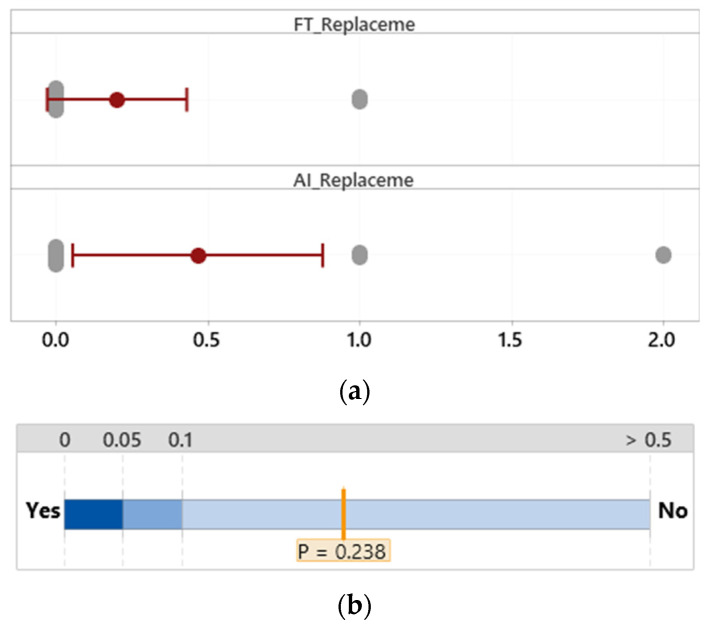
Mean difference in unnecessary replacement. (**a**) Distribution of data. (**b**) Two-sample mean *t*-test.

Unnecessary replacement may occur when a participant incorrectly diagnoses a component, leading them to replace components that are already in proper working condition. Within the AId-system group, out of the 15 participants, 5 participants made seven unnecessary replacements, whereas in the FTd-system group, out of the 15 participants, 3 participants made three unnecessary replacements. However, the difference between the two groups in the number of unnecessary replacements was found to be statistically insignificant (*p* = 0.238), as illustrated in Figure 11.

### 3.3. Additional Experiment

**Figure 12:**
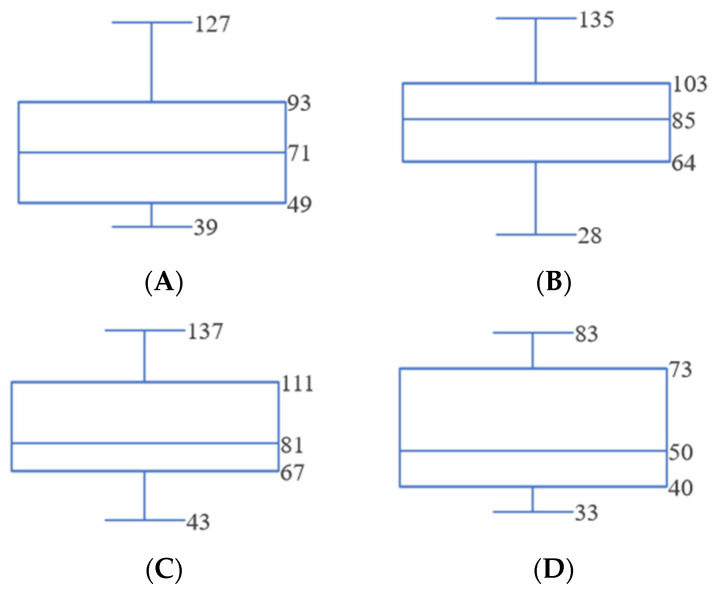
Preliminary study result of additional participants (units in seconds). (**A**) Sensor diagnosis time. (**B**) Battery diagnosis time. (**C**) Light bulb Diagnosis time. (**D**) Wire diagnosis time.

An additional 15 participants (mean age = 26.20 years old, range 19–36) were recruited to complete the same tasks in the same setting, but without any diagnosis support system, i.e., neither the FTd system nor the AId system was used in these experiments. This additional experimental result helps to identify the impact of the diagnosis support systems during the maintenance activity. The average times to diagnose the sensor, the battery, the light bulb, and the wire of newly recruited participants were 75 s, 85 s, 86 s, and 54 s, respectively, as shown in Figure 12. To observe how the usage of a diagnosis support system affected technicians during the maintenance activities by comparing three groups, one-way ANOVA analysis was conducted.

#### 3.3.1. Time to Complete

**Figure 13:**
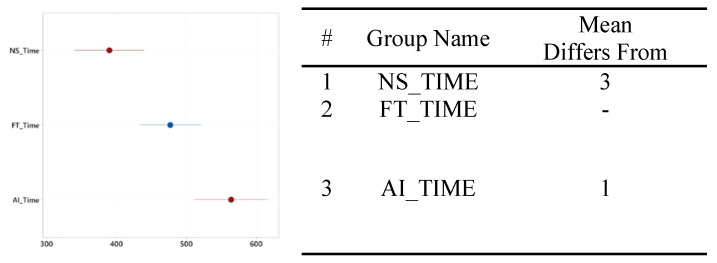
One-way ANOVA analysis for time to complete.

**Figure 14:**
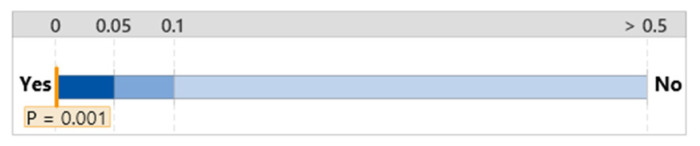
Significance test for mean time to complete difference.

The group that did not use either type of diagnosis support system took 389.87 s on average to complete the maintenance task, with a standard deviation of 110.52. The group without the diagnosis support system took the least amount of time, and the group that used the AId-system took the most time to complete the task, as shown in Figure 13. A statistically significant difference only existed between the AId-system group and the group that did not use a diagnosis support system, as shown in Figure 14.

#### 3.3.2. NASA TLX

**Figure 15:**
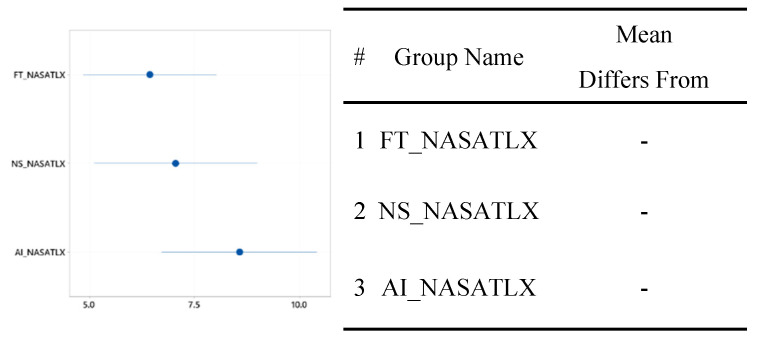
One-way ANOVA analysis for NASA TLX.

**Figure 16:**
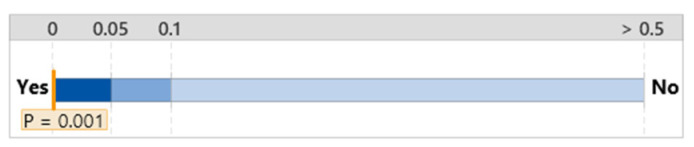
Significant test for NASA TLX difference.

The mean workload of the group without the diagnosis support system was 7.06 with a standard deviation of 4.27; this workload was greater than that of the group that used the FTd-system, but less than that of the group that used the AId-system, as shown in Figure 15. Although the mean workload of the group that used the AId-system was slightly greater than the other two groups’ mean workloads, a statistically significant difference between the three groups’ workloads was not identified according to the ANOVA analysis, as shown in Figure 16.

#### 3.3.3. Maintenance Attempt

The expected number of attempts for the group that did not use any diagnosis support system can be estimated using negative hyper-geometric distribution, since it is a similar process to the “trial-and-error” process [50] and it utilized eight attempts; this number of attempts could also be achieved when the other groups strictly followed the recommendations of the support systems and made no errors. The mean number of maintenance attempts of the group that did not use the support systems was 6.60 attempts, with a standard deviation of 1.92. This is fewer than the analytical expectation and the other groups.

**Figure 17:**
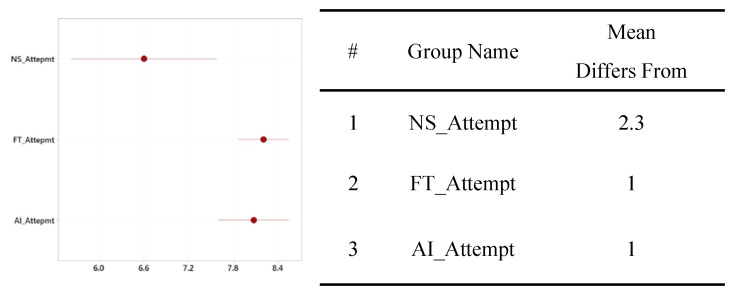
One-way ANOVA analysis for diagnosis attempt.

**Figure 18:**
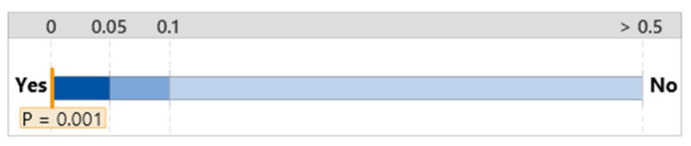
Significance test for diagnosis attempt.

There was a statistical difference in the mean number of maintenance attempts between groups. The mean of the group that did not use the diagnosis support system was lower than the other groups’ means, as shown in Figure 17 and Figure 18.

#### 3.3.4. Unnecessary Replacement

**Figure 19:**
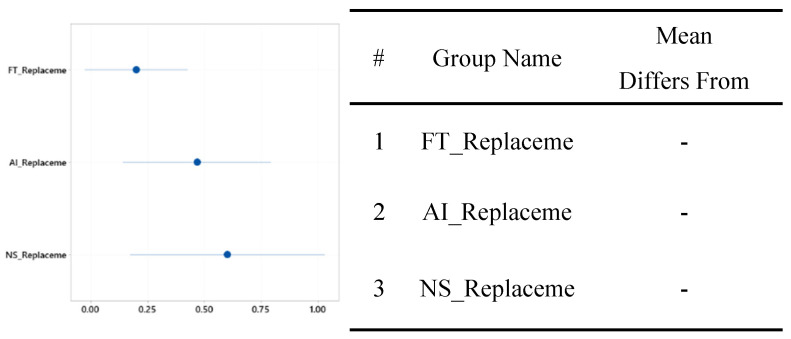
One-way ANOVA analysis for unnecessary replacement.

**Figure 20:**
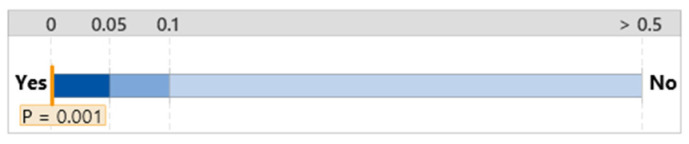
Significance test for unnecessary replacement.

The mean of unnecessary replacement for the group that did not use the diagnosis support system was 0.6, with a standard deviation of 0.91. Again, the mean for this group was slightly greater than that of the other two groups, as shown in Figure 19. However, there were no statically significant differences between the means of the three groups, as shown in Figure 20, according to the one-way ANOVA analysis.

## 4. Interaction Analysis by NGOMSL Model

Based on the results of the experiments, no differences were observed in the workloads of technicians or unnecessary replacement between groups. However, the statistically significant difference in the metrics related to maintenance speed was as shown in Table 5. In order to better understand these results, the task was modeled using an established approach to study human–computer interactions and identify possible ways to reduce the cognitive load on a technician. Specifically, the natural GOMS language (NGOMSL), an extension of the goals, operators, methods, and selection rules (GOMS) model, is used. GOMS is “a description of the knowledge that a user must have in order to carry out tasks on a device or system” [51]. And NGOMSL is “in program form and provides predictions of operator sequence, execution time, and time to learn the methods” [52]. NGOMSL provides an abstraction for modeling the task at the key-stroke level and provides estimates of demands on cognitive load by estimating the peak memory and working memory requirements of the modeled task.

The experiment settings were modeled using NGOMSL for further analysis. First, the difference in “time to complete” was investigated. Next, the effect of the number of options shown in the AId-system was studied.

### 4.1. The Difference in Time to Complete

Time to complete mainly depended on two factors [50]: the number of maintenance attempts and the time spent on diagnosing, repairing, or replacing a component.

**Figure 21:**
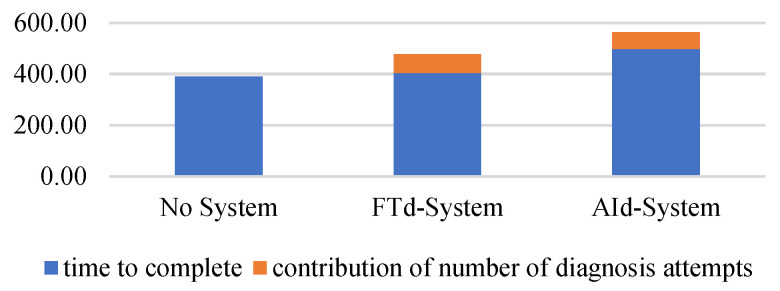
Difference in time to complete between groups.

If the number of diagnosis attempts is significantly different between groups, the mean time to complete should be different. Every additional attempt to diagnose a wire takes 45 s, and an attempt to diagnose a sensor takes 66 s according to the preliminary study session results of this experiment. Since there was a difference of approximately 1.5 diagnosis attempts between the group without a diagnosis support system and that with a diagnosis support system, the contribution of the difference in attempts was about 68 s, as shown in Figure 21.

**Figure 22:**
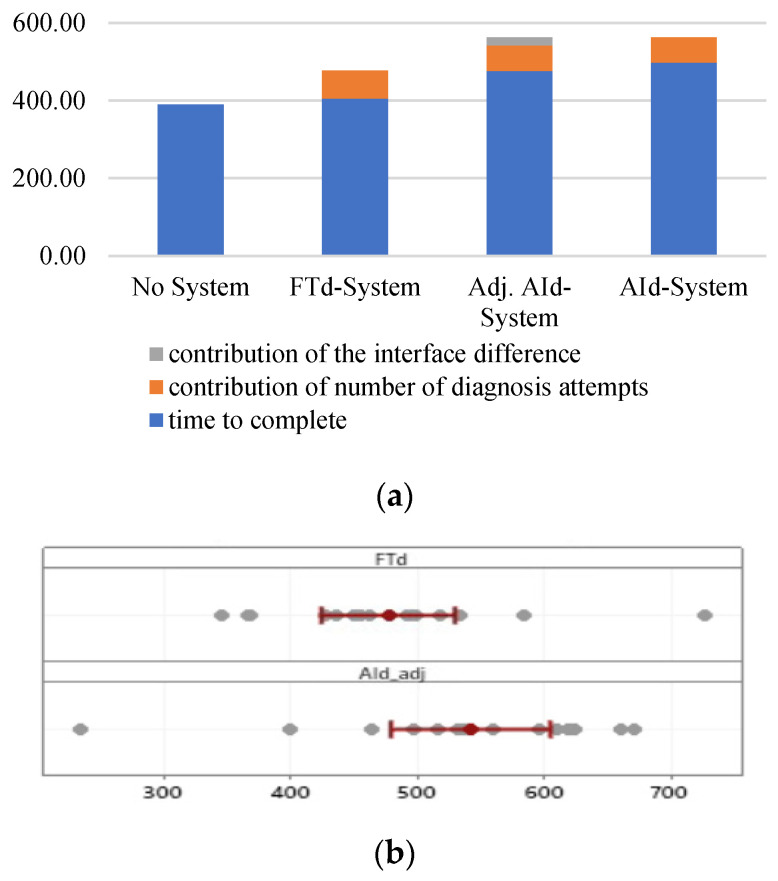
Average time to complete difference (units in seconds). (**a**) Average time to complete difference. (**b**) Distribution of data.

To understand the difference in completion times between groups that used a diagnosis support system, the interface difference was evaluated by comparing NGOMSL statements. For every diagnosis attempt, the interaction with the AId-system requires an additional four NGOMSL statements, which contribute roughly 2.65 s according to the keystroke level model [53,54], as shown in Table 6. If this difference is adjusted, the average time to complete maintenance for the AId-system becomes 542.09 s. After the adjustment, the two groups’ difference in the average time to complete is 65 s; the difference is not statistically significant (*p*-value 0.1), as shown in Figure 22.

### 4.2. Impact of the Number of Options Presenting in AId-System

In the current experimental setting, the AId-system showed all possible options at the same time with the corresponding probabilities, which may have demanded a larger cognitive load. The effect of the number of options shown in the AId-system was analyzed using two metrics: the peak load on working memory and the working memory density. The interaction between the user and the system was modeled using NGOMSL; this model was composed of eight methods and two selection rules, as shown in Table 7.

Under the current experimental setting, participants detected faulty components at the 1st, 4th, 7th, and 8th diagnosis attempts when they strictly followed the recommendation of the system. This processing sequence required a total of 321 NGOMSL statements and a total of 1429 items to be remembered. In this case, the peak working memory and working memory density for this setting were 7 and 4.45, respectively.

If the AId-system only showed a limited number of options, such as two, three, or four options at every diagnosis attempt, the difference occurred in “Method to accomplish the goal of ‘select a component’”. For example, if there were nine options to be shown, it would require twenty-six NGOML statements, whereas only five NGOML statements would be required if only two options were shown, as shown in Table 8.

The total number of NGOML statements, the total number of items to be remembered, the peak working memory, and the working memory density were compared for the different settings, as shown in Table 9.

**Figure 23:**
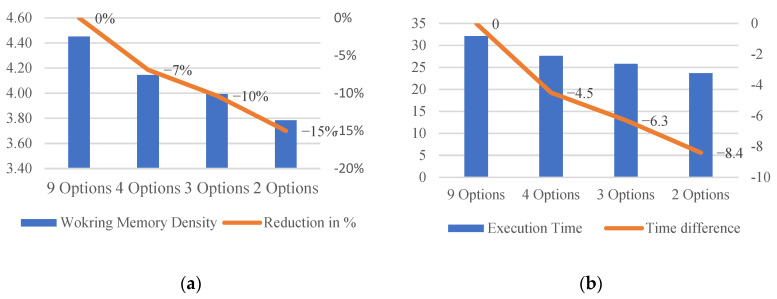
The difference in working memory density and execution time due to the number of options shown. (**a**) Working memory density. (**b**) NGOMSL statement execution time.

When the number of options shown in the AId-system is limited, the peak working memory does not change. However, as the number of options shown decreases, the execution time and the working memory density also decrease. The estimation shows that if the number of options shown were limited to two, the execution time and cognitive load would be reduced by 8.4 s and 15%, respectively, as shown in Figure 23.

The AId-system, when tested with human subjects, did not perform as well as the FTd-system. On further investigation using NGOMSL, it has been found that the specific user interface used in the AId-system requires more keystrokes and presents more options, resulting in a higher cognitive load by requiring higher working memory density. Future versions of smart diagnosis support systems can be expected to perform better through improvements in the user interface that reduce the need for working memory density.

## 5. Conclusions and Future Work

One challenge in adopting new systems and changing the current process is not only the budget, but also unknown outcomes. The effect of introducing smart systems needs to be analyzed from multiple perspectives and in the context of real and practical usage [6]. Although much effort has been made to study tools, methodologies, and enablers for offering smart service–product systems [1], more studies are required for social impact and to have an effect on users. Thus, this research is motivated to investigate the unwelcomed effects of a “smart” system when it aids the technician during maintenance and to develop a human-centered diagnosis support system for maintenance as a first step.

### 5.1. Discussion

In summary, the experiment results and NGOMSL analysis of this experiment show that adopting a smart diagnosis support system can potentially improve maintenance productivity by reducing the number of maintenance attempts without burdening technicians with new work. However, to further reduce the working memory density of technicians who interact with AId-systems, one needs to consider delivering limited information to the technician when they design the AId-system.

The experiment results show that the two different diagnosis support systems do not have statistically significant impacts on the workload of technicians or on the maintenance quality. On the other hand, the participants’ maintenance time is statistically different depending on the system with which they interact.

The effect of the interface differences in the two systems was analyzed using the NGOMSL model. Interacting with the AId-system required extra keystrokes compared to interacting with the FTd-system. Also, for every diagnosis attempt, the AId-system required an extra 2.65 s, which is one of the main sources of the maintenance time difference between the two systems. Although, when the adjustment was made, the difference in time to complete was statistically insignificant (*p*-value 0.1), further study is required since the *p*-value was small and there were limitations of the experimental settings, such as sample size and population group (college students). Additionally, the effect of the number of options shown in the AId-system was analyzed. As a smaller number of options were shown to the user, a smaller cognitive load was imposed during maintenance. The estimation showed that the maintenance time and the cognitive load could be reduced by 8.4 s and 15% if only two options were shown to participants. However, further experimentation is required in order to confirm the estimation of cognitive load reduction and its impact.

### 5.2. Future Work

Nevertheless, although the experiment in this paper attempted to duplicate a real CNC machine maintenance environment in the lab, the results may not be the same as the results of an experiment conducted at a real site with a real machine. Further studies are necessary with more realistic settings to completely capture the effects of diagnosis support systems on technicians during maintenance. Also, additional studies are warranted in the future with different maintenance tasks, since the experimental result differs based on the machine that needs to be maintained and the skill of technicians.

## Figures and Tables

**Table 1:** Machine learning application in maintenance.

Algorithm	Reference	Year	Usage and Application
Bayesian Network (BN)	Nguyen et al. [16]	2015	Localizing the root cause of machine failure
	Yang and Lee [19]	2012	Prognostic maintenance for a semi-conductor machine
	Correa et al. [24]	2009	Identifying the quality issue in a machining process
	Abu-Samah et al. [31]	2015	Prognostic maintenance: predicting the degradation of components
Neural Network (NN)	Wu et al. [18]	2019	Detecting machine setup problem
	Hong et al. [20]	2012	Detecting semi-conductor machine setup problem and classifying faults
	Barakat et al. [21]	2011	Prognostic maintenance
	Zhang et al. [25]	2013	Prognostic maintenance: predicting the degradation of components in a machine
	Yu et al. [27]	2009	Prognostic maintenance: classifying the fault depending on the signal
	Biswal and Sabareesh [28]	2015	Prognostic maintenance: predicting the degradation level of the wind turbine components
Support Vector Machine (SVM)	Demetgul [22]	2013	Identifying the cause of a didactic modular production system failure
	Kumar et al. [23]	2016	Prognostic maintenance for a steel plate manufacturing facility
	Hsueh and Yang [26]	2009	Prognostic maintenance: predicting the degradation of a milling machine
Other	Amihai et al. [29]	2018	Prognostic maintenance: predicting the condition of the industrial pump
	Kolokas et al. [30]	2018	Prognostic maintenance: predicting the down time of an industrial machine

**Table 2:** Proximity sensor model setting.

Real Machine Setting	Model Setting	Quantity in the Model	Broken
Sensor	Sensor	1	No
Power	Battery	1	Yes
Power Switch	Switch	1	Yes
Sensor Connection	Signal Cable to Light Bulb	1	Yes
Cables	Cables	4	No
I/O Board	Light bulb	1	Yes

**Table 3:** Maintenance log example.

Log	System	Sensor	Switch	Power	LB	YW	RW	GW	WW	BW
1	f	g	b	g	g	g	g	g	g	g
2	f	g	b	g	g	g	g	g	g	g
3	f	g	g	b	g	g	g	g	g	g
…										
*n*	f	g	g	b	b	g	g	g	g	b

f = fail, g = in good condition, and b = in bad condition.

**Table 4:** Cause of failure probability calculation example.

Description of Conditions	Count	Probability
P (switch = b|system = f and others = g)	10	14%
P (sensor = b|system = f and others = g)	2	3%
P (Power = b|system = f and others = g)	9	13%
P (LB = b|system = f and others = g)	8	11%
P (YW = b|system = f and others = g)	3	4%
P (RW = b|system = f and others = g)	2	3%
P (GW = b|system = f and others = g)	4	6%
P (WW = b|system = f and others = g)	3	4%
P (BW = b|system = f and others = g)	7	10%
P (more than 2 components = b|system = f)	22	31%

**Table 5:** Summary of experimental results.

	FTd-System	AId-System	No Support System
Time to Complete	477.20 s	563.47 s	389.87 s
NASA TLX	6.44	8.56	7.06
Diagnosis Attempt	8.20 attempts	8.07 attempts	6.60 attempts
Unnecessary Replacement	0.20 parts	0.46 parts	0.60 parts

**Table 6:** Keystroke level model analysis on the interface difference.

**FT Diagnosis Support System**			
**#**	**Type**	**Statement**	**Time**
1	$t_{p}$	Read Screen (Diagnosis Instruction)	0.10
2	-	Do Diagnosis	-
3	P	Move the mouse to the “NO” Button	1.10
4	B	Click the “YES” Button	0.10
**AI** **Diagnosis Support System**			
**#**	**Type**	**Statement**	**Time**
E1	$t_{p}$	Read Screen	0.10
E2	M	Select a component (Mental)	1.35
E3	P	Move the mouse to the selected component	1.10
E4	B	Click the selected component	0.10
1	$t_{p}$	Read Screen (Diagnosis Instruction)	0.10
2	-	Do Diagnosis	-
3	P	Move the mouse to the “NO” Button	1.10
4	B	Click the “YES” Button	0.10

**Table 7:** NGOMSL for AId-System.

**1. Method to “maintain a system”**	
S1	Retrieve LTM tat current item in command sequence is maintain a system
S2	Accomplish the goal of “diagnose a component”
S3	Decide: if the system is not working,
S4	then Goto 1-S2
S5	Report goal of ‘maintain a system’ accomplish
**2. Method to accomplish the goal of “diagnose a component”**	
S1	Recall “diagnose a component” and accomplish the goal of “select a component”
S2	Recall A and accomplish the goal of “click a component”
S3	Read screen and retain instruction
S4	DO (External—diagnosing selected component)
S5	Forget instruction and accomplish the goal of “repair a component”
S6	Report goal of “diagnose a component” accomplish
**3. Method to accomplish the goal of “select a component”**	
S1	read next button in a screen, retain the component name as A, and retain probability as B
S2	read next button in a screen, retain the component name as A’ and retain probability as B’
S3	Decide if B’ > B
S4	then retain B’ as B, A’ as A and forget A’ and B’
S5	forget B and report the goal of “select a component” accomplish
**4. Method to accomplish the goal of “click a component”**	
S1	Read screen
S2	Move the cursor to A
S3	Click A
S4	forget A and report the goal of “click a component” accomplish
**5. Method to accomplish the goal of “select a component”**	
S1	Read screen
S2	Move the mouse to “Yes”
S3	Click “Yes”
S4	report goal of “report status” and “repair a component” accomplish
**6. Method to accomplish the goal of “fix a component”**	
S1	Read screen
S2	Move the mouse to “No”
S3	Click “No”
S4	read screen and retain instruction
S5	DO (External—replacing a component)
S6	Forget instruction and accomplish the goal of “report system condition”
**7. Method to accomplish the goal of “finalize the maintenance”**	
S1	read screen
S2	move the mouse to “Yes”
S3	Click “yes”
S4	report goal of “finalize the maintenance”, “report system condition”, “fix a component”, and goal of “repair a component” accomplish
**8. Method to accomplish the goal of “update the status”**	
S1	Read screen
S2	Move the mouse to “No”
S3	Click “No”
S4	report goal of “update the status”, “report system condition”, “fix a component”, and “repair a component” accomplish
**SR1. Selection rule set for the goal of “repair a component”**	
	if the component is in good condition, then accomplish the goal of “report status”
	if the component is not in good condition, then accomplish the goal of “fix a component”
**SR2. Selection rule set for the goal of “report system condition”**	
	if the system condition is good then accomplish the goal of ”finalize the maintenance”
	if the system condition is not good then accomplish the goal of “update the status”

**Table 8:** NGOML statements for two difference cases.

**When 9 options are shown**		
**#**	**Ref**	**Statement**
1	1-s1	retrieve LTM that current item in command sequence is “maintain a system”
2	1-s2	Accomplish goal of “diagnose a component”
3	2-s1	Recall “diagnose a component” and accomplish the goal of “select a component”
4	3-s1	read next button in a screen, retain the component name as A, and retain probability as B
5	3s-2	read next button in a screen, retain the component name as A’ and retain probability as B’
6	3-s3	Decide if B’ > B
7	3-s4	then retain B’ as B, A’ as A and forget A’ and B’
Statements 5 through 7 would be executed 7 more times		
29	3-s5	forget B and report the goal of “select a component” accomplish
**When** **2 options are shown**		
**#**	**Ref**	**Statement**
1	1-s1	retrieve LTM that current item in command sequence is “maintain a system”
2	1-s2	Accomplish goal of “diagnose a component”
3	2-s1	Recall “diagnose a component” and accomplish the goal of “select a component”
4	3-s1	read next button in a screen, retain the component name as A, and retain probability as B
5	3s-2	read next button in a screen, retain the component name as A’ and retain probability as B’
6	3-s3	Decide if B’ > B
7	3-s4	then retain B’ as B, A’ as A and forget A’ and B’
8	3-s5	forget B and report the goal of “select a component” accomplish

**Table 9:** Number of NOGML statements difference.

# Options	# of Statements	# of Items to Be Remembered	Peak Working Memory	Working Memory Density
9 options	321	1429	7	4.45
4 options	276	1144	7	4.14
3 options	258	1030	7	3.99
2 options	237	897	7	3.78

## Data Availability

The raw data supporting the conclusions of this article will be made available by the authors on request.

## Appendix A

Videos on Proximity Switch (27 February 2024): https://www.youtube.com/watch?v=hr2o_F79gLE; https://youtu.be/iLWCZfuY65A?si=e-PFxtrwxmx6qtRf.
